# Integrated Serum Metabolome and Gut Microbiome to Decipher Chicken Amino Acid Improvements Induced by Medium-Chain Monoglycerides

**DOI:** 10.3390/metabo13020208

**Published:** 2023-01-30

**Authors:** Tao Liu, Shengyue Ruan, Qiufen Mo, Minjie Zhao, Jing Wang, Zhangying Ye, Li Chen, Fengqin Feng

**Affiliations:** 1 College of Biosystems Engineering and Food Science, Ningbo Research Institute, Zhejiang University, Hangzhou 310058, China; 2 Hangzhou Longyu Biotechnology Co., Ltd., Hangzhou 310003, China; 3 The Institute of Animal Husbandry and Veterinary Science, Zhejiang Academy of Agricultural Sciences, Hangzhou 310021, China

**Keywords:** chicken amino acid, medium-chain monoglycerides, broiler chickens, serum metabolome, gut microbiome

## Abstract

Chicken muscle yield and amino acid composition improvements with medium-chain monoglyceride (MG) supplementation were reported by previous studies, but the underlying mechanism was uncertain. This study aimed to decipher chicken amino acid improvements induced by medium-chain monoglycerides in the views of metabolomics, gene expression, and the gut microbiome. Newly hatched chicks (12,000 chicks) were weighed and randomly divided into two flocks, each with six replicates (1000 chicks per replicate), and fed a basal diet (the control group, CON) or a basal diet enriched with 300 mg/kg MG (the treated group, MG). Results demonstrated that MGs significantly increased the chicken flavor and essential and total amino acids. The serum amino acids and derivatives (betaine, l-leucine, l-glutamine, 1-methylhistide), as well as amino acid metabolism pathways in chickens, were enhanced by MG supplementation. Gene expression analysis exhibited that dietary MGs could improve muscle protein synthesis and cell growth via the mTOR/S6K1 pathway. Dietary MGs enhanced the cecal amino acid metabolism by selectively increasing the proportion of genera *Lachnospiraceae_NK4A136_group* and *Bacteroides*. Conclusively, the present study demonstrated that dietary MGs improved chicken amino acid composition via increasing both gut amino acid utilization and muscle amino acid deposition.

## 1. Introduction

In the last two decades, metabolomics has been widely used to study low molecular weight metabolites (<1000 daltons), providing us with information about metabolite profiles and integrated metabolic pathways of farmed animals in response to nutritional intervention [1]. Alterations in the circulating metabolites profile can partially reflect the influences of nutritional treatments on energy and nutrient metabolism, among them, some key metabolites are identified to be closely related to animal performance and meat quality. For instance, l-glutamine is the richest amino acid in both the bloodstream and the body’s free amino acid pool. Broilers fed diets containing l-glutamine present improved productive performance and better meat quality [2,3,4]. As an essential amino acid, higher plasma leucine can significantly stimulate muscle protein synthesis by enhancing translation initiation factor activation in neonatal pigs [5,6]. Higher levels of betaine exert a positive influence on both animal performance (average daily gain and feed nutrients utilization) and carcass yield [7]. Currently, mass spectrometry (MS)-based and non-MS-based techniques such as nuclear magnetic resonance (NMR) are the two main types of platforms that have been widely used for metabolomic studies [8]. MS-based metabolomics is widely used in tissues, biofluids, or cells due to the lipophilicity and polarity of metabolites.

Medium-chain monoglycerides (MGs) are a group of saturated 8–12 carbon fatty acids monoglycerides containing glycerol monolaurate (GML), glycerol monodecanoate (GMD), and glycerol monocaprylin (GMC), showing broad antibacterial spectrum and strong synergistic antimicrobial activity in vivo and in vitro [9,10,11,12]. Recently, numerous studies demonstrated that dietary supplementation with MGs modulates the structure and function of gut microbiota and exhibits a close relationship with chicken production and quality. Junhong declares that feed additive GML can promote *Lachnospiraceae*, *Christensenellaceae*, and *Ruminococcaceae* colonization in the chicken cecum, thereby increasing the short-chain fatty acids (SCFAs) content and improving the chicken body weight [13]. Kong et al. [14] state that dietary GML selectively increases the abundance of *Lachnospiraceae*, *Faecalibacterium*, and *Bacteroides*, which is found to be positively related to a lowered feed conversion rate (FCR) in broiler chickens [15]. Chickens fed diets containing a GML and GMD mixture are selectively enriched with an unclassified genus of the *Lachnospiraceae* family, *Bifidobacteriaceae*, and *Bacteroides* [16], along with improved average body weight, muscle amino acid, and carcass yield [17]. Intestinal microecology has proven to be the main functional target of dietary GML ameliorating metabolic syndrome and obesity in mice, as GML-mediated metabolic improvements are all abolished after the addition of antibiotics [18,19].

In addition, 16S rRNA gene sequencing combined with MS-based metabolomics reveals that dietary butyrate glycerides modulate lipid metabolism and energy homeostasis in broilers through increasing *Bifidobacterium* and bacterial metabolites [20]. Dietary lauric acid modulates gut microbiota (*Faecalibacterium*, *Ruminococcaceae_UCG-014*) and serum metabolites (lysophosphatidylcholines and phosphatidylcholines) to enhance immune functions, suppress inflammation, and modulate lipid metabolism of broilers [21]. These findings offer us an effective way to directly investigate the relationship between dietary intervention and meat quality. Moreover, our previous study reported that dietary MG-induced muscle amino acid changes were closely related to the increased relative abundance of an unclassified genus of *Lachnospiraceae*, *Bifidobacteriaceae*, *Bacteroides,* and bacterial amino acid metabolism gene in experimentally reared broilers [16,17]. In another study, the content and metabolism pathways of muscle amino acids were significantly improved with dietary MGs in large-scaled broilers [9]. However, the influences and linkages of related serum metabolism and key gut microbiota that were responsible for muscle amino acid and yield were not uncovered. The present study was conducted to integrally reveal chicken amino acid improvements induced by medium-chain monoglycerides using combined approaches of 16S rRNA sequencing, quantitative PCR assays, and MS-based metabolomics in large-scaled broilers.

## 2. Materials and Methods

### 2.1. Animal Management and Diets

Newly hatched chicks (male Chinese indigenous, yellow-feathered broiler breeds) were weighed and randomly divided into two flocks, each with six replicates (1000 chicks per replicate). Chicks were raised on the ground and fed and drunk *ad libitum* on a modern farm with 24 h constant lighting for a 70-day experiment. The broiler chicks were fed a basal diet (the control group, CON) or a basal diet enriched with 300 mg/kg MG (the treated group, MG). The basal diet was formulated referring to previous work in Table S1 [17]. MG is a mixture of GML and GMD that is produced by Hangzhou Longyu Biotechnology Co., Ltd. (Zhejiang, China).

Feed consumption and the number and corresponding body weight of dead birds were recorded daily for each replicate to calculate and adjust the feed conversion rate of broilers throughout the entire experiment. All of the chickens were weighed with replicates at 0 and 70 days of age. Body weight (BW), average daily gain (ADG), average feed intake (FI), and feed conversion rate (FCR) were calculated subsequently.

### 2.2. Sample Collection

After fasting for 12 h, two randomly selected chickens out of a replicate were sacrificed in the morning at 71 days of age. Blood was drawn from the wing veins and the serum was obtained using centrifugation (2000× *g*, 15 min, 4 °C). Pectoralis major and cecal digesta was promptly isolated and frozen [22]. All samples were kept at −80 °C before analysis.

### 2.3. Muscle Amino Acid Determination

The method of muscle amino acid measurement refers to a previous study [17]. The dried muscle samples were dissolved in 6M HCl and digested at 150 °C for 2 h under a pure nitrogen atmosphere. Then, the digested samples were derived with reagent (ethanol: water: triethylamine: phenyl-isothiocyanate, 7:1:1:1, *v*/*v*/*v*/*v*). The amino acid composition was measured using HPLC (Waters e2695; Waters Corporation, Milford, MA, USA) equipped with an Ultimate AQ-C18 column.

### 2.4. Serum Untargeted Metabolomics

#### 2.4.1. Extraction of Serum Metabolites

Serum (100 μL) from each chicken (*n* = 6) was added into 2.0 mL tubes containing a cold methanol/acetonitrile solution (1:1, *v/v*, 400 μL). After 60 s vortex, all tubes were left standing at −20 °C for 1 h; thereafter, the supernatant was separated (15,000× *g*, 4 °C, 20 min) and freeze-dried. The dried samples were separately redissolved in an acetonitrile/water solution (1:1, *v/v*, 100 μL) before analysis.

#### 2.4.2. Mass Spectrometry Analysis

The serum metabolites profile was separated using an ACQUITY UPLC HSS T3 column (1.8 µm, 2.1 mm × 100 mm, Waters, Milford, MA, USA) and an ACQUITY UPLC BEH Amide column (1.7 µm, 2.1 mm × 100 mm, Waters, Milford, MA, USA) on liquid chromatography (1290 Infinity UHPLC, Agilent, Santa Clara, CA, USA). The mobile phase consisted of A (25 mM ammonium hydroxide and ammonium acetate in water) and B (100% acetonitrile). The gradient elution procedure was: 0–1 min, 95% B; 1–14 min, B linearly reduced to 65%; 14–16 min, B linearly reduced to 40%; 16–18 min, 40% B; 18–18.1 min, B linearly increased to 95%; 18.1–23 min, 95% B. The mass spectrometric data were acquired with a time-of-flight mass spectrometer (Triple TOF 5600/6600, AB SCIEX, Framingham, MA, USA). The Electrospray ionization source conditions and operating conditions referred to a previous study [9].

The raw MS data processing referred to previous works [9,23]. Principal component analysis (PCA) and orthogonal partial least-squares discriminant analysis (OPLS-DA) were performed to reveal the metabolic alterations between groups. The VIP value of each metabolite was used to reflect its contribution to the classification. The metabolites with VIP values larger than 1 were further applied to measure their significance using the Student’s *t*-test. The significantly different metabolites were blasted against with the KEGG database to retrieve the KOs and corresponding pathways. The significantly different pathways were screened based on Fisher’s exact test.

### 2.5. Quantitative PCR Analysis

The mRNAs of pectoralis major were extracted using reagent Kits following the instructions (Invitrogen, Waltham, MA, USA). The total RNA of each sample was reverse-transcribed using the PrimeScript^TM^RT Kit (Takara, Kyoto, Japan). Gene expression was analyzed in the Roche Light Cycler 480 system (Indianapolis, IN, USA) using Takara TB Green *Premix* Ex Taq^TM^ (Kyoto, Japan). The reference gene was Glyceraldehyde-3-phosphate dehydrogenase, and the results were calculated relative to the CON group using the ^2–ΔΔ^Ct method [24]. Primer sequences of the genes were listed in Table S2.

### 2.6. Gut Microbiome

The DNA of cecal digesta was extracted using QIAGEN Kits referring to instructions (Venlo, The Netherlands). After concentration determination, the DNA content of each sample was diluted to 1 ng/μL. Then, the V3−V4 region of the 16S rRNA gene was amplified with PCR using primers 338F and 806R. The amplicons were purified and quantified before they were sequenced (HiSeq2500 platform, San Diego, CA, USA).

Clean reads were obtained after assembly (FLASH software v1.2.11) and quality control (QIIME v1.9.1) of raw paired-end reads. Then the operational taxonomic units (sequences ≥ 97% similarity) were picked, and taxonomic information was annotated using the UPARSE v 7.0.1009 and Silva128/16S_bacteria database [25]. Microbial composition, α-diversity (Shannon, Simpson, Chao, and Ace), and β-diversity (principal coordinates analysis (PCoA) based on unweighted unifrac) were analyzed. Predictive bacterial function profiling was performed using PICRUSt (http://picrust.github.io/picrust). The changes in Orthologue (KOs) and pathways were identified using the KEGG database. The characteristic taxa and function were revealed with the LEfSe (http://huttenhower.sph.harvard.edu/galaxy/) algorithm and showed with STAMP (version 2.1.3) [16]. The raw data have been uploaded to NCBI (PRJNA638502).

### 2.7. Statistical Analysis

The student’s *t*-test and Wilcoxon rank-sum test were used to screen for significant differences. Differences were expressed as * *p* < 0.05, ** *p* < 0.01, and *** *p* < 0.001. A *p*-value greater than 0.05 but less than 0.1 was discussed as tendencies.

## 3. Results

### 3.1. Chicken Productive Performance

Compared to the CON group, the BW, FI, and ADG were 74.26 g, 110.11 g, and 1.05 g higher in the MG group (Table 1), respectively, while the FCR decreased by 1.08%.

### 3.2. Dietary MG Alters Muscle Amino Acids Content

The muscle glutamic acid, proline, serine, leucine, glycine, phenylalanine, alanine, tyrosine, methionine, and threonine content were increased with MG supplementation (*p* < 0.05, Table S3). The dietary MG increased (*p* < 0.05) the total and essential amino acids by 7.14% and 7.40%, respectively.

### 3.3. Chicken Serum Metabolome

**Figure 1:**
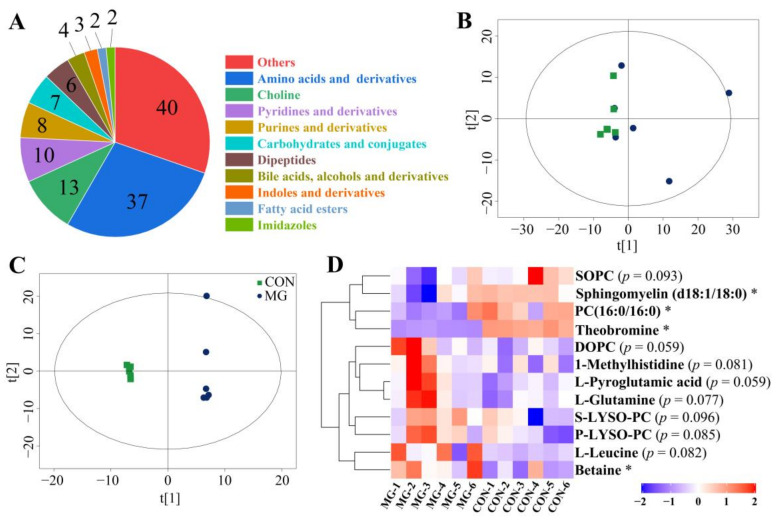
Serum metabolite differences with MG supplementation. (**A**) Pie chart of serum metabolites. Principal component analysis (PCA) score plot (**B**) and OPLS-DA score plot (**C**). (**D**) Heatmap of significant differential metabolites (VIP value > 1, *p*-value < 0.1). SOPC, 1-Stearoyl-2-oleoyl-sn-glycerol 3-phosphocholine; PC (16:0/16:0), phosphatidylcholine (16:0/16:0); DOPC, 1,2-dioleoyl-sn-glycero-3-phosphatidylcholine; S-LYSO-PC, 1-Stearoyl-2-hydroxy-sn-glycero-3-phosphocholine; P-LYSO-PC, 1-Palmitoyl-sn-glycero-3-phosphocholine. Chicks fed a basal diet (the control group, CON) or a basal diet enriched with 300 mg/kg MG (MG). Data are shown as mean ± SD (*n* = 6, * *p* < 0.05).

A total of 132 serum metabolites were identified out of 9157 acquired ion peaks. Derivatives (37), choline (13), pyridines and derivatives (10), purines and derivations (8), carbohydrates and conjugates (7), and dipeptides were the major chicken serum metabolites (6, Figure 1A). The principal component analysis (PCA) scores plot showed obvious clustering and drift according to MG treatment with a slight overlap (Figure 1B). The OPLS-DA scores plot showed intensive clustering in the MG group and a distinct shift away from the CON group (Figure 1C), indicating that the chicken serum metabolic profiles differed a lot. Moreover, the R^2^ and Q^2^ values in the permutation test were 0.999 and 0.694, respectively, showing that the model had credible cumulative interpretation and predictive ability. Twelve differential serum metabolites were selected using a VIP value over 1 and a *p*-value less than 0.1 in this model including four down-regulated metabolites and eight up-regulated metabolites that were responsible for the serum profile differences (Figure 1D).

**Figure 2:**
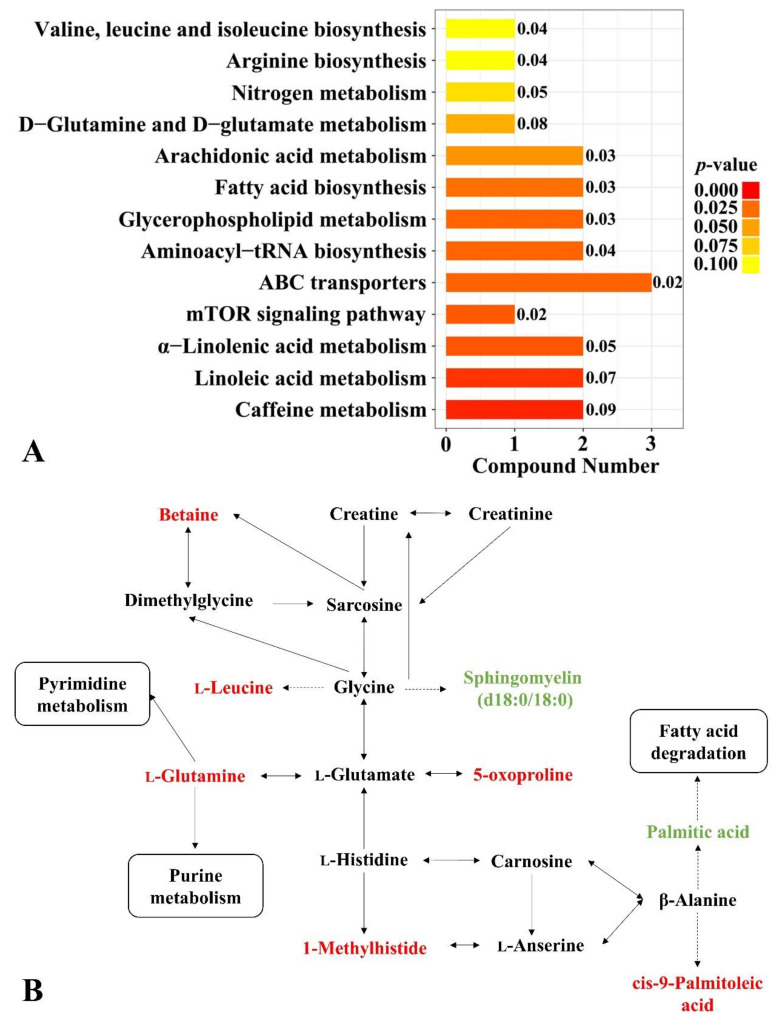
Changes in metabolic pathways and serum metabolites with MG supplementation. (**A**) differential serum metabolic pathways (compound number were the total count of metabolites related to metabolic pathways); (**B**) metabolites in red and green were up-regulated and down-regulated, respectively.

A total of thirteen metabolic pathways were notably influenced by MG addition (Figure 2A), including five lipid metabolism pathways (linoleic acid metabolism, α-linolenic acid metabolism, glycerophospholipid metabolism, fatty acid biosynthesis, arachidonic acid metabolism), three amino acid metabolism pathways (d-glutamine and d-glutamate metabolism, arginine biosynthesis, valine, leucine, and isoleucine biosynthesis), one energy metabolism pathway (nitrogen metabolism) and another four metabolism pathways. Pathways involving in the chicken serum metabolite differences by dietary MG were summarized and sketched in Figure 2B according to the KEGG database, mainly involving amino acid metabolism pathways (valine, leucine and isoleucine biosynthesis, glycine, serine and threonine metabolism, histidine metabolism, valine, leucine, and isoleucine degradation, d-glutamine and d-glutamate metabolism) and lipid metabolism pathways (fatty acid degradation, sphingolipid metabolism, fatty acid elongation), as well as seven key differential serum metabolites related to MG supplementation (betaine, l-leucine, l-glutamine, 1-methylhistide, sphingomyelin (d18:1/18:0), and PC (16:0/16:0)).

### 3.4. Dietary MG Affects the mRNA Expression of Muscle Growth Regulation

**Figure 3:**
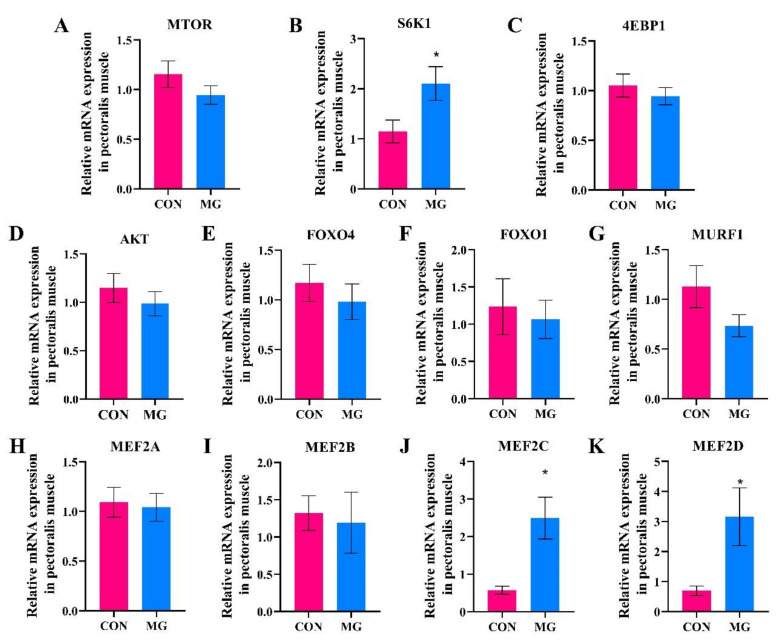
Changes in mRNA expression of muscle protein synthesis and myocyte differentiation with MG supplementation. (**A**), Mammalian target of rapamycin; (**B**), Ribosomal p70 S6 Kinase; (**C**), Eukaryotic translation initiation factor 4E-binding protein 1; (**D**), Serine/threonine protein kinase B; (**E**), Forkhead box O4; (**F**), Forkhead box protein O1; (**G**), Muscle RING-finger protein-1; (**H**), Myocyte enhancer factor 2A; (**I**), Myocyte enhancer factor 2B; (**J**), Myocyte enhancer factor 2C; (**K**), Myocyte enhancer factor 2D. Chicks fed a basal diet (the control group, CON) or a basal diet enriched with 300 mg/kg MG (MG). Data are shown as mean ± SD (*n* = 12, * *p* < 0.05).

The relative expression of S6K1, MEF2C, and MEF2D increased by 83.52%, 336.48%, and 353.54% in the MG group, respectively (*p* < 0.05, Figure 3B,J,K).

### 3.5. Dietary MG Affects the Chicken Gut Microbiota

#### 3.5.1. Changes in Chicken Microbial Diversity and Structure

**Figure 4:**
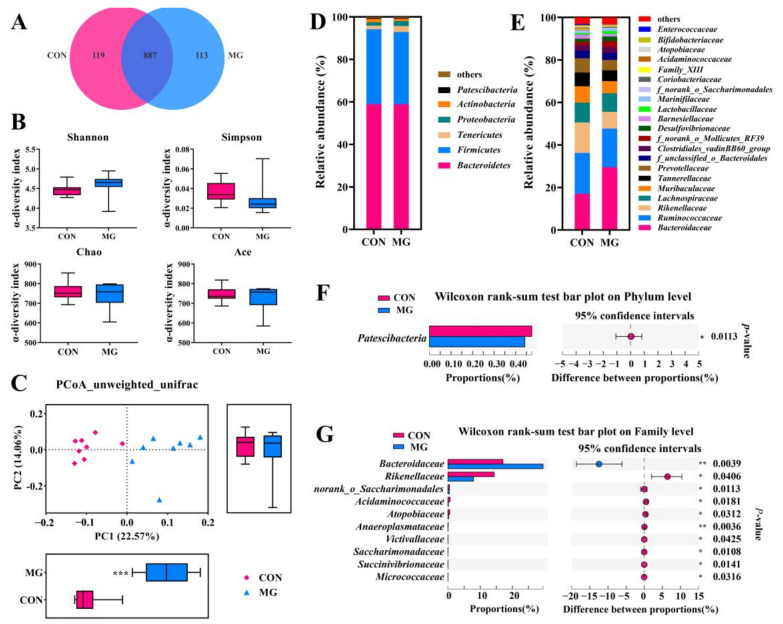
Changes in chicken gut microbiota with MG supplementation. (**A**) OTUs Venn diagram. (**B**) α-diversity (Shannon, Simpson, Chao, and Ace). (**C**) Principal coordinates analysis (PCoA). Microbial taxonomic profiling at the phylum (**D**) and family (**E**) level. Relative abundance of differential phyla (**F**) and families (**G**). Chicks fed a basal diet (the control group, CON) or a basal diet enriched with 300 mg/kg MG (MG). Data shown as mean ± SD (*n* = 8, * *p* < 0.05, ** *p* < 0.01, *** *p* < 0.001).

The current study obtained 1119 OTUs, and 119 and 113 OTUs only existed in the CON and MG groups (Figure 4A), respectively. The dietary MG did not exert changes on the ACE, Chao, Simpson, and Shannon index (Figure 4B). However, the principal coordinates analysis (PCoA) plot showed intensive clusters in the MG group and distinct drift away from the CON group without any overlaps. Moreover, the distance between the MG and CON group in the PC1 coordinates represented significant differences with an explanation of 22.57% observed total variance (*p* < 0.001, Figure 4C), suggesting a distinct variation of the gut microbiota community. Taxonomic profiling demonstrated that cecal flora was dominated by *Bacteroidetes*, followed by *Firmicutes*, *Tenericutes*, *Proteobacteria*, *Actinobacteria,* and *Patescibacteria* (Figure 4D), and the relative content of *Patescibacteria* showed significant differences (Figure 4F). *Bacteroidaceae*, *Ruminococcaceae*, *Rikenellaceae*, *Lachnospiraceae*, *Muribaculaceae*, *Tannerellaceae*, *Prevotellaceae*, an unclassified family of *Bacteroidales* order, and *Clostridiales_vadinBB60_group* were the main families of phyla *Bacteroidetes* and *Firmicutes* (Figure 4E). At the family level (Figure 4E), increased *Bacteroidaceae* and decreased *Rikenellaceae* were found in the MG group.

**Figure 5:**
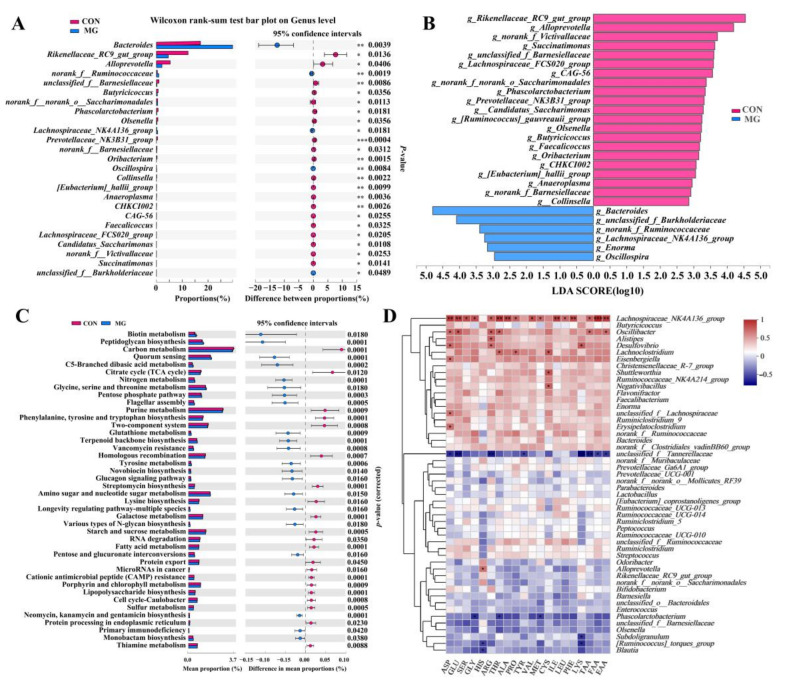
Changes in chicken gut microbiota and predicted function with MG supplementation. (**A**) Relative abundance of differential genera. (**B**) Differential microbial genus (LDA > 2.0, *p* < 0.05). (**C**) Predicted microbial functions. (**D**) Correlation between chicken cecal microbiota composition and muscle amino acids. TAA, all detected amino acids. Muscle amino acid was obtained from a previous study [9]. * *p* < 0.05, ** *p* < 0.01, *** *p* < 0.001.

Dietary MG increased (*p* < 0.05) the abundance of *Bacteroides*, a no rank genus of the *Ruminococcaceae* family, *Lachnospiraceae_NK4A136_group*, *Oscillospira*, and an unclassified genus of *Burkholdderiaceae* and reduced (*p* < 0.05) the content of *Rikenellaceae_RC9_gut_group*, *Alloprevotella*, an unclassified genus of *Barnesiellaceae*, *Butyricicoccus*, a no rank genus of the *Saccharimonadales* order, *Phascolarctobacterium*, *Olsenella*, *Prevotellaceae_NK3B31_group*, an unclassified genus of *Barnesiellaceae*, *Oribacterium*, *Collinsella*, *[Eubacterium]_hallii_group*, *Anaeroplasma*, *CHKCI002*, *CAG_56*, *Faecalicoccus*, *Lachnospiraceae_FCS020_group*, *Candidatus_Saccharimonas*, a no rank genus of *Victivallaceae*, and *Succinatimonas* (Figure 5A). LEfSe revealed 27 specific bacterial genera (Figure 5B). *Rikenellaceae_RC9_gut_group*, *Alloprevotella*, a no rank genus of *Victivallaceae*, *Succinatimonas*, an unclassified genus of *Barnesiellaceae*, *Lachnospiraceae_FCS020_group*, *CAG_56*, a no rank genus of the *Saccharimonadales* order, *Phascolarctobacterium*, *Prevotellaceae_NK3B31_group*, *Candidatus_Saccharimonas*, *[Ruminococcus]_gauvreauii_group*, *Collinsella*, *Butyricicoccus*, *Faecalicoccus*, *Oribacterium*, *CHKCI002*, *[Eubacterium]_hallii_group*, *Anaeroplasma*, an unclassified genus of *Barnesiellaceae*, and *Olsenella* were rich in the CON group. (Figure 5B). *Bacteroides*, an unclassified genus of *Burkholdderiaceae*, a no rank genus of the *Ruminococcaceae* family, *Lachnospiraceae_NK4A136_group*, *Enorma*, and *Oscillospira* were abundant in the MG group (Figure 5B).

#### 3.5.2. Changes in Chicken Gut Microbiota Community and Function

The predictive functions of the microbial genes belonging to carbohydrate and amino acid metabolism were significantly changed with dietary MG (Figure 5C). The dietary MG significantly increased the gene abundances of four carbohydrate metabolism pathways (amino sugar and nucleotide sugar metabolism (*p* = 0.015), the pentose phosphate pathway (*p* = 0.0003), pentose and glucuronate interconversions (*p* = 0.016), and C5-branched dibasic acid metabolism (*p* = 0.0002)) and three amino acid metabolism pathways (tyrosine metabolism (*p* = 0.0006), glutathione metabolism (*p* = 0.0009), and glycine, serine, and threonine metabolism (*p* = 0.018)). The dietary MG also depleted the microbial gene abundance of carbohydrate metabolism (starch and sucrose metabolism (*p* = 0.0005), citrate cycle (TCA cycle, (*p* = 0.012), and galactose metabolism (*p* = 0.0001)) and amino acid metabolism (lysine biosynthesis (*p* = 0.016) and phenylalanine, tyrosine, and tryptophan biosynthesis (*p* = 0.0001)).

#### 3.5.3. Correlation of Chicken Gut Microbiota Composition with Amino Acid Content

Spearman’s correlation analysis was conducted to reveal the relationship between the chicken gut microbiota composition (at the genus level) and amino acid content (Figure 5D). A total of 16 genera out of the top 50 were closely related to chicken amino acids. Among them, 11 genera were positively (*p* < 0.05) related to chicken amino acid content, but 4 genera showed a notable negative relationship (*p* < 0.05). Moreover, *Lachnospiraceae_NK4A136_group*, the significantly increased genera in the MG group, were positively (*p* < 0.05) related to chicken amino acid content. The increased *Bacteroides* represented a notably positive relationship with muscle amino acid, but no significant differences were observed. However, the decreased *Phascolarctobacterium* were negatively (*p* < 0.05) correlated with chicken amino acid content. The results suggested the alterations in chicken amino acids were closely correlated to the changes in gut microbiota composition with thee dietary MG.

## 4. Discussion

Muscle protein is the main component in raw poultry meat (18.4–23.4%), playing a key role in poultry meat quality evaluation [26]. Amino acids are the basic building blocks of muscle proteins, and their composition and relative quantity are important indexes for meat quality evaluation [17]. Dietary MGs significantly increased the content of 10 muscle amino acids including both flavor taste and essential amino acids in the current study, enhancing the umami and sweet taste, as well as the nutritional value of chicken. These results were consistent with a previous study where muscle aspartic acid, serine, proline, glutamic acid, and arginine content were increased in chickens fed 300 mg/kg MG [17]. The inclusion of 300 mg/kg GML was also reported to increase chicken muscle serine, glycine, arginine, histidine, tyrosine, threonine, methionine, phenylalanine, and lysine content [27]. Consistent with previous studies where both the body weight and carcass yield were improved with MG supplementation [17,27], the dietary MG notably increased the chicken body weight and daily weight gain in the current study. Similarly, a couple of recently published studies reported that dietary medium-chain monoglycerides increased chicken body weight in both yellow-feathered and white-feathered broilers [11,13,28,29]. These findings indicated that the increased muscle amino acid content induced with MG supplementation did not only improve the chicken meat quality, but also the main contributions to improved chicken muscle mass and body weight.

In this research, betaine, l-leucine, l-glutamine, 1-methylhistide, sphingomyelin (d18:1/18:0), and PC (16:0/16:0) were screened as chicken serum metabolites in response to dietary MG supplementation. Betaine is a trimethyl derivative of glycine and has amino acid properties, and it is widely distributed in most organisms and could participate in protein metabolism by donating its methyl group [7,30,31]. Betaine was reported to be involved in regulating chicken growth, nutrient metabolism, and antioxidant balance, which was recognized as a “carcass modifier” due to its lipotropic and growth-promoting effects [32]. Chen et al. [30,32] stated that dietary betaine improved chicken productive performance and lean meat yield. The higher serum betaine level provides us with biochemical evidence of dietary MGs improving the chicken growth performance and carcass yield. l-Glutamine is the richest amino acid in both the bloodstream and the body’s free amino acid pool [2,3], which plays a vital role in body ammonia balance due to its two ammonia groups that can accept excess ammonia and release it when needed to form biologically important molecules such as amino acids, nucleotides, protein, etc. Broilers receiving diets containing l-glutamine showed better performance and meat quality [2,3,4]. The up-regulated serum l-glutamine in the current study indicated that MG supplementation may improve the muscle amino acid content and meat quality of broilers by boosting the amino acid utilization and deposition [9]. Escobar et al. [5] and Duan et al. [6] stated that increased plasma leucine could stimulate muscle protein synthesis by enhancing translation initiation factor activation in neonatal pigs [5,6], suggesting that dietary MGs may promote the muscle protein deposition of broilers through up-regulating the serum l-leucine level. In addition, 1-Methylhistide was the end-product of histidine metabolism and the important precursor for the synthesis of anserine, which exerts a significant impact on the nutritional values, antioxidant capacity, and the umami taste of meat [33,34]. In the present study, the up-regulated serum 1-methylhistide in the MG group reflected the higher level of muscle anserine that has been confirmed in our previous study [9]. Similar to Wu et al. [21], the content changes of serum phosphatidylcholine (including PC (16:0/16:0), SOPC, sphingomyelin (d18:1/18:0), and S-LYSO-PC, P-LYSO-PC) implied that inclusion of MGs affected chicken lipid metabolism, but their effects on muscle fat content and mass were limited [9,17]. The metabolic pathway analysis offered an integrated and direct connection of metabolites by the reconstruction of a biochemical reaction network [35,36]. The dietary MG supplementation enhanced the chicken amino acids and lipid metabolism pathways which was reflected in the KEGG enrichment analysis. Moreover, serum metabolites are the end-products of physiological processes caused by body states or by exposure to environmental factors or drugs, which can provide an integrated view of the biochemical environment of both body fluid and tissue. Therefore, the enhanced amino acid metabolic pathways and related metabolites revealed the chicken body’s amino acid improvement in the MG-treated group.

Our previous study showed that both the composition and metabolism pathways of muscle amino acids in broilers were altered in response to MG supplementation [9]. Results obtained with serum untargeted metabolomics in the current study reminded us that the muscle protein synthesis in the pectoralis major may be affected by MG supplementation. Protein synthesis and proteolysis were regulated by the mTOR signaling pathway in avain [37,38,39], in which the phosphorylation of 4E binding protein (4EBP1) and 70 kDa ribosomal protein S6 kinase (S6K1) by mTOR activation initiated mRNA translation and protein synthesis. Higher expression of S6K1 in the MG group indicated that the dietary MG promoted muscle protein synthesis via the mTOR/S6K1 pathway in the present study, resulting in improved skeletal muscle mass and carcass yield [17]. Moreover, the up-regulated serum leucine was recognized as the activator of the mTOR/S6K1 pathway, as the up-regulated effects of branched-chain amino acids on the mTOR/S6K1 pathway have been well demonstrated [40,41,42]. The unchanged relative expression of AKT, FOXO4, FOXO1, and MURF1 demonstrated that the dietary MG exerted no effects on muscle protein proteolysis [39,43]. Myocyte enhancer factor 2 family (MEF2) including MEF2A, MEF2B, MEF2C, and MEF2D, play multiple roles (regulating the differentiation, maintenance, and regeneration of muscle cells) in muscle cells to regulate myogenesis and morphogenesis [44,45]. Wen et al. [46] demonstrated that dietary methionine improved breast muscle growth and carcass yield of commercial broilers with increased mRNA levels of MEF2A and MEF2B. Chen et al. [30] stated that betaine (up-regulated in serum in the present study) supplementation enhanced muscle growth with increased MEF2B expression in breast muscle. Therefore, the significantly higher expression of MEF2C and MEF2D indicated that MG supplementation may also promote muscle growth and performance by improving the growth and development of muscle cells.

Numerous studies proved that intestinal microecology is the main functional target of MG supplementation exerting benefit effects on animals [16,18,19]. Similarly, dietary supplementation of MGs altered the cecal microbiota profile of broilers with increases in the family *Bacteroidaceae* and decreases in the family *Rikenellaceae* in this study. Similar to our previous study, broilers receiving the 300–600 mg/kg MG-supplemented diet showed a distinct variation of gut microbiota structure and a notably higher family of *Bacteroidaceae* in cecum compared with the CON group [16]. The supplementation of graded levels of single GML (450 and 600 mg/kg) in diets also exerted significant alterations on both the structure and composition of chicken gut microbiota [27]. Kong et al. [14] reported notable different gut microbiota profiles and improved microbial diversity in broilers fed diets containing 300, 600, 900, or 1200 mg/kg GML at both 7 and 14 days of age. Lan et al. [13] stated that the dietary supplementation of GML significantly modulated the gut microbiota community of broilers at both 28 and 56 days of age.

Specifically, at the genus level, dietary MGs increased the content of chicken cecal *Bacteroides*, a no rank genus of the *Ruminococcaceae* family, and *Lachnospiraceae_NK4A136_group*. Similar to our previous study, the relative abundance of an unclassified genus of the *Lachnospiraceae* family, *Bacteroides,* and *Bifidobacteriaceae* in cage-reared chickens was increased [16]. Dietary supplementation of GML selectively increased the proportion of an unclassified genus of the *Lachnospiraceae* family and *Bifidobacteriaceae* in broilers [27]. It has been reported that the abundance of *Lachnospiraceae_FE2018_group* and *Bacteroides* in chicks was increased with the addition of graded levels of GML (300, 600, 900, and 1200 mg/kg) [14]. Similarly, supplementation of lauric acid to the basal diet increased the colonization of *Bacteroides* and *Lactobacillus* in lipopolysaccharide-challenged broilers [47]. In summary, these findings revealed that dietary supplementation of medium-chain monoglycerides increased the colonization of *Lachnospiraceae* and *Bacteroides*, which belong to the *Firmicutes* and *Bacteroidetes* phylum, respectively. It has been reported that *Bacteroides* play a vital role in the breakdown of complex molecules, especially in the utilization of nitrogenous substances by the host and the gut microbiota [48]. The increased *Bacteroides* were probably responsible for improved protein digestibility in this study, which was increased from 51.40 to 54.49% after MG supplementation (data not shown). *Lachnospiraceae* can utilize complex plant-derived carbohydrates, in particular, they readily degrade less recalcitrant indigestible polysaccharides and starch to release sugars for both the gut microbiota and host [49]. Apajalahti et al. [50] and Singh et al. [15] stated that a higher relative abundance of *Lachnospiraceae* was closely related to the increased body weight of commercial broilers chickens, suggesting increased *Lachnospiraceae_NK4A136_group* may contribute to the improved productive performance in the present study. Additionally, similar to a previous study [16], the increased *Lachnospiraceae_NK4A136_group* and *Bacteroides* were positively correlated with muscle amino acid. Moreover, the bacterial gene abundance of the carbohydrate and amino acid metabolism pathways by PICRUSt function prediction was significantly increased with MG supplementation. Therefore, the increase of feed protein and carbohydrate utilization efficiency in the gut by selectively increasing the proportion of *Lachnospiraceae_NK4A136_group* and *Bacteroides* may partially explain the chicken amino acid improvements.

## 5. Conclusions

The present study demonstrated that the enhancement of feed protein digestion and absorption with 300 mg/kg MG supplementation mainly started from the gut microbiota modulation (*Lachnospiraceae_NK4A136_group* and *Bacteroides*). Moreover, we observed remarkably increased serum amino acids and derivatives (betaine, l-leucine, l-glutamine, 1-methylhistide), as well as enhanced amino acid pathways in the serum after MG supplementation. Coincidentally, the chicken amino acid composition and gene expression of chicken protein synthesis were improved after MG treatment. Conclusively, the present study partially explained that the dietary MG improved the chicken amino acid composition by increasing amino acid utilization in the gut microbiota, serum, and muscle. The current study offered us a new approach to control chicken quality in future poultry production.

## Figures and Tables

**Table 1:** Chicken productive performance of large-scaled broilers.

	CON	MG	*p* Value
Average daily gain (ADG), g	35.36 ± 0.31	36.41 ± 0.30 ***	<0.001
Average feed intake (FI), g	6028.08 ± 8.43	6138.19 ± 8.75 **	0.006
Body weight (BW), g	2515.20 ± 20.30	2589.46 ± 21.59 ***	<0.001
Feed conversion rate (FCR)	2.399 ± 0.021	2.373 ± 0.020	0.084

CON, the control group. MG, the MG-containing group. Differences were expressed as ** *p* < 0.01 and *** *p* < 0.001.

## Data Availability

The data presented in this study are available in the article and Supplementary Materials.

## Supplementary Materials

The following supporting information can be downloaded at: https://www.mdpi.com/article/10.3390/metabo13020208/s1, Table S1: Composition and nutrient level of the basal diet for starter (1 to 28 d) and finisher (29 to 70 d) phase of broilers; Table S2: The sequence of primers; Table S3: Dietary MG affects chicken amino acid content.
