# Supplementary material for: Integrated Serum Metabolome and Gut Microbiome to Decipher Chicken Amino Acid Improvements Induced by Medium-Chain Monoglycerides
Source: Metabolites. 2023 Jan 30;13(2):208. doi: 10.3390/metabo13020208 (PMC9966585; doi:10.3390/metabo13020208)
Supplement: Supplementary file 1 [file metabolites-13-00208-s001.zip › metabolites-2088744-supplementary.pdf]

metabolites

MDPI

Table S1. Composition and nutrient level of the basal diet for starter (1 to 28 d) and finisher (29 to 70 d) phase of broilers.

	Starter (day 1 to 28)	Finisher (day 29 to 56)
Ingredients, %		
Corn	54.7	60.5
Wheat	5	5
Soybean meal	29	23
Corn gluten meal	6	6
Soybean oil	1.0	1.5
Dicalcium phosphate	1.7	1.5
Limestone	1.3	1.2
Sodium chloride	0.30	0.30
Vitamin-mineral premix[1]	1.00	1.00
Calculated composition, %		
Metabolizable energy (MJ/kg)	12.17	12.55
Lysine	1.1	0.95
Methionine	0.45	0.40
Analyzed composition, %		
Crude protein	20.96	18.89
Calcium	0.99	0.89
Total phosphorus	0.66	0.60
Methionine + cystine	0.85	0.72

[1]Supplied per kilogram of diet: Vitamin A 9600 IU, Vitamin D 2700 IU, Vitamin E 36 mg, Vitamin K_3 3.00 mg, Vitamin B_1 3.00 mg, Vitamin B_2 10.5 mg, Vitamin B_6 4.2 mg, nicotinic acid 60.00 mg, D-calcium pantothenate 18 mg, folic acid 1.50 mg, biotin 0.225 mg, $ZnSO_4{\cdot}H_2O$ 212.52 mg, $CuSO_4{\cdot}H_2O$ 33.18 mg, $FeSO_4{\cdot}H_2O$ 247.75 mg, $MnSO_4$ .H_2O 263.98 mg, $Ca(IO_3)_2$ 85.80 mg, Na_2SeO_3 37.60 mg.

Table S2. The sequence of primers.

Name	GeneBank ID	Sequence (5′-3′)
GAPDH, Glyceraldehyde-3-phosphate dehydrogenase	NM_204305.1	F: ACTGTCAAGGCTGAGAACGG<br>R: AGCTGAGGGAGCTGAGATGA
mTOR, Mammalian target of rapamycin	XM_417614.4	F: AGTGAGAGTGATGCGGAGAG<br>R: GAAACCTTGGACAGCGGG
S6K1, Ribosomal p70 S6 Kinase	NM_001030721.1	F: GGTGGAGTTTGGGGGCATTA<br>R: GAAGAACGGGTGAGCCTAA
4EBP1, Eukaryotic translation initiation factor 4E-binding protein 1	XM_424384.4	F: GCGAATGTAGGTGAAGAAGAGC<br>R: GGCTGGTGGGAATCCTCAAA
Akt, Serine/threonine protein kinase B	NM_001005838.1	F: CATTCCCGCCATTATGAATGAAGTA<br>R: CTTGTAGCCAATGAATGTGCCATC
FOXO1, Forkhead box protein O1	NM_204328.1	F: CATAACCAGCCAACACCTGC<br>R: AATTCCCACCCTTCCGTAGC
FOXO4, Forkhead box O4	XM_426261	F: CTCGCTAAGGTCAGAAGTAAA<br>R: TCCTCAGTCACGGTTGGT
MURF1, Muscle RING-finger protein-1	XM_424369.4	F: GAACCTGCTGGTGGGAGAACA

		R: GTGCTCCCCCTTCTTGAGTG
MEF2A, Myocyte enhancer factor 2A	NM_204864	F: TCACGAGAATAATGGACGAA<br>R: TGCTGGCATACTGAAAGAGT
MEF2B, Myocyte enhancer factor 2B	XM_430389	F: CACAGCCTCACTGGTTTCCC<br>R: GACACCCGCTCTGACTTGATG
MEF2C, Myocyte enhancer factor 2C	XM_001231661	F: TGAGCGTAACAGACAGGTGAC<br>R: GCGTAAGAGGAGTCAGGATCG
MEF2D, Myocyte enhancer factor 2D	NM_001031600	F: GAGCGTGTTGTGCGACTG<br>R: GCTGCCTTTACGGTATTTATCC

Table S3. Dietary MG affects chicken amino acid content.

g/100g dry material	CON	MG
Aspartic acid	7.186 ± 0.785	7.675 ± 0.551
Glutamic acid	5.902 ± 0.572	6.364 ± 0.316*
Serine	2.921 ± 0.256	3.132 ± 0.123*
Glycine	3.620 ± 0.312	3.934 ± 0.106**
Alanine	4.734 ± 0.411	5.074 ± 0.227*
EAA		
Lysine	6.172 ± 0.815	6.824 ± 0.751
Methionine	2.469 ± 0.216	2.661 ± 0.118*
Threonine	3.532 ± 0.300	3.744 ± 0.128*
Leucine	6.716 ± 0.627	7.180 ± 0.304*
Isoleucine	4.261 ± 0.391	4.548 ± 0.265
Arginine	5.086 ± 0.630	5.553 ± 0.505
Valine	4.105 ± 0.437	4.338 ± 0.364
Phenylalanine	3.419 ± 0.301	3.650 ± 0.164*
Histidine	3.454 ± 0.358	3.542 ± 0.298
Σ EAA	30.675 ± 2.588	32.946 ± 1.561*
Proline	2.997 ± 0.248	3.254 ± 0.097**
Tyrosine	3.216 ± 0.266	3.437 ± 0.154*
Cysteine	0.641 ± 0.120	0.697 ± 0.130
Σ AA	70.566 ± 6.241	75.607 ± 3.514*

Chicken amino acid content was published in a previous study [9]. Chicks fed a basal diet (the control group, CON) or a basal diet enriched with 300 mg/kg MG (MG). Σ EAA, all detected essential amino acids; Σ AA, all detected amino acids. Data expressed as mean ± SD (n = 12, ** p < 0.01).
